# Double-anonymised peer review: a new option for authors at *Chemical Science*†

**DOI:** 10.1039/d1sc90122b

**Published:** 2021-06-29

## Abstract

Introducing double-anonymised peer review in *Chemical Science*. Graphical abstract image adapted from © Shutterstock/M-SUR.
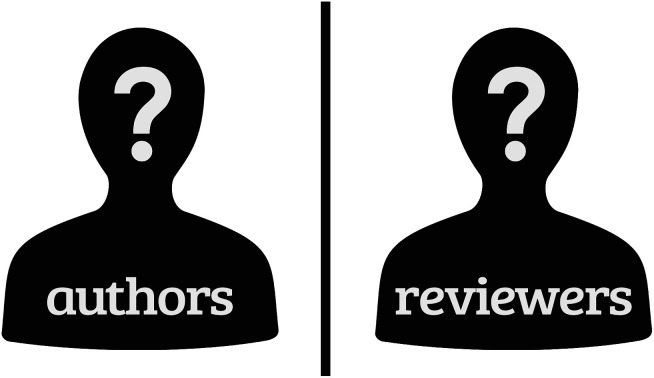

We are pleased to announce that authors will now be able to select the option of double-anonymised peer review for their manuscript on submission to *Chemical Science*. For the majority of journals at the Royal Society of Chemistry, and across the chemical sciences more widely, peer review is carried out using a traditional single-anonymised approach, where reviewers are anonymous but author names and affiliations are known to reviewers. Double-anonymised peer review takes this one step further and conceals the authors’ and reviewers’ identities from each other.

Members of the *Chemical Science* readership will likely be familiar with the term ‘double-blind peer review’ and how it operates. In 2020, the STM Association, a global trade association for academic and professional publishers, formed a working group to look at peer review taxonomy to develop and recommend standardised terminology to make peer review practices transparent and inclusive for all.^1^ It was decided that the use of the word ‘blind’ in this context, as well as being easy to misinterpret, is ableist in origin, *i.e.* arises from and reinforces stereotypical understandings of disability which impact negatively on blind and low vision people. Therefore, this taxonomy recommends that the term ‘double-blind’ is replaced by ‘double-anonymised’. As we introduce this model on *Chemical Science*, we will also be extending the terminology more widely across all Royal Society of Chemistry journals that offer it.

## Why are we introducing double-anonymised peer review?

The Royal Society of Chemistry is committed to tackling the significant gender, racial, socioeconomic and geographical challenges that affect the scholarly publishing process. In recent years we have studied our own publishing operation to identify and quantify gender bias^2^ and initiated a joint commitment with 38 other publishing organisations to set new standards within scholarly publishing to enable a more inclusive and diverse culture.^3^ Bringing the option of double-anonymised peer review to *Chemical Science* is another step towards a fairer and more inclusive process.

Since the launch of *Chemical Science*, the only peer review model that has been available to our authors is the more traditional single-anonymised approach. While we continue to trust in the effectiveness of that system, we also want to respond to the growing number of requests for alternative models that are coming from the broad and diverse chemical sciences community.

In July 2017, one of our sister journals, *ChemComm*, trialled the use of double-anonymised peer review and permanently adopted it a year later. Their experience identified that 10% of their authors chose this approach, with an above average number of these authors being resident in India and the Middle East. The quality of the reviews and author satisfaction were comparable for both single- and double-anonymised routes.^4^ Since then our *Environmental Science* family of journals has also taken the move to offer the option of double-anonymised peer review to their authors.^5^ Other science, technical and medical publishers have also trialled and adopted double-anonymised peer review recently, for example the Society of Environmental Toxicology and Chemistry journals published by Wiley^6^ and the Institute of Physics, who have implemented double-anonymised peer review across all of their journals.^7,8^

By offering double-anonymised peer review we hope to give *Chemical Science* authors more choice and control over how their manuscript is handled. For any author who feels that they may be unfairly subject to a bias in relation to their gender, ethnicity, career stage, affiliation or otherwise, we hope this option will allow them to have greater trust in the peer review of their manuscript. Over time, this will also provide data that will help us as a journal and a publisher to identify sources of bias so that we can take further steps to eliminate them.

While there are clear strengths to this approach for reducing bias, we must also recognise the challenges of double-anonymised peer review.^9^ Under this model, the onus to ensure a manuscript is appropriately anonymised will lie with the authors, and we appreciate the difficulties attached to this. As so much scientific research draws on previous work and has iterative components, appropriately referencing a manuscript may leave a clear trail to the authors’ identities. Moreover, as the use of preprint servers such as ChemRxiv increases, a reviewer might easily identify the authors should they choose to preprint their manuscript.

Another challenge is time. Anonymising a manuscript requires a different writing style and this may have an increased time cost for authors, for example if they have previously submitted the work to a different journal that offers only single-anonymised peer review. We feel that these challenges are more than compensated by the additional choice that this system offers for authors.

Critics of double-anonymised models that are optional to authors might argue cases of positive bias are still possible where the single-anonymised route is chosen. We recognise that this approach alone will not tackle all sources of bias within the publishing system. Our Associate Editors will continue to have oversight of both author and reviewer identities. Working together we can help to reduce bias, regardless of the peer review model used. We are also working to further increase the diversity of both our Editorial Board and reviewer pool and to increase the breadth of perspectives that we draw upon for editorial decisions. Offering the option of double anonymised peer review has the potential to reduce bias with respect to gender, race and ethnicity, country of origin, or affiliation, and we see it as an important step in our continuing drive towards fairness and inclusivity.

## How does double-anonymised peer review fit with Open Science practices

Alongside *Chemical Science*’s diamond Open Access credentials, the journal has also been taking steps to adopt wider Open Science practices, such as making author contribution statements mandatory^10,11^ and more strongly encouraging data availability statements.^11,12^ While at first glance double-anonymised peer review could be seen to be making the peer review process more opaque, we view it as complementary to more open peer review models. Double-anonymised peer review aims to reduce the bias during the review process. Open and transparent peer review models, conversely, work to increase the visibility of the decision-making process after review, so that readers can understand the steps taken during the peer review process that led to the publication of an article. Both double-anonymised and open or transparent peer review models can operate at the same time to bring fairness and transparency to the publication process. While *Chemical Science* does not offer open or transparent peer review at this time, should we introduce this to the journal in future, we feel these two options for authors can work well in tandem with each other.

## How will double-anonymised peer review work in practice for our authors and reviewers?

As an author, you will be able to choose whether your manuscript undergoes double-anonymised peer review or traditional, single-anonymised peer review. Both options will be available to all authors and your preference can be selected during the submission process. If you choose the double-anonymised peer review option, you should ensure that your manuscript and all associated files are suitably anonymised before submission; please refer to our checklist when preparing your manuscript files (see ESI†). It is the responsibility of the author to ensure that their manuscript is suitably anonymised.

As a reviewer, you will receive an invitation where the identity of the authors is kept confidential for papers where the authors have chosen the option of double-anonymised peer review. All further communication will omit author name and affiliation details. If you determine the identity of the authors for manuscripts where double-anonymised peer review was chosen, we would ask you to continue with your review, focusing on the suitability of the manuscript for the *Chemical Science* audience in line with our reviewing procedure. However, we would ask you to please highlight in the confidential comments to the Editor that you were able to identify the authors when submitting your review.

We look forward to working with the authors and reviewers of *Chemical Science* with the inclusion of this new review option.

 

May Copsey

Executive Editor, *Chemical Science*

 

Jeremy Allen

Deputy Editor, *Chemical Science*

 

Andrew I Cooper

Editor-in-Chief, *Chemical Science*, & Department of Chemistry, University of Liverpool, UK

## Supplementary Material

SC-012-D1SC90122B-s001
