# Supplementary material for: Double-anonymised peer review: a new option for authors at Chemical Science
Source: Chem Sci. 2021 Jun 29;12(25):8586–8. doi: 10.1039/d1sc90122b (PMC8246075; doi:10.1039/d1sc90122b)
Supplement: SC-012-D1SC90122B-s001 [file SC-012-D1SC90122B-s001.pdf]

Electronic Supplementary Material (ESI) for Chemical Science.
This journal is © The Royal Society of Chemistry 2021

ROYAL SOCIETY OF CHEMISTRY

Checklist when preparing your submission for double-anonymised peer review

Please note that it is your responsibility as an author to anonymise your manuscript prior to submission.
• The author list and affiliations should be removed from both your main article and any supplementary files. Please also remove the acknowledgements and author contribution sections from your main article.
• Your cover letter and justification should have author and affiliation details removed.
• Make sure that you suitably anonymise all files to remove the author or user name in the document properties (this information can be removed in both pdf and Microsoft Word files by navigating to: File / Properties).
• Check that your figures and schemes do not include author details.
• Avoid phrasing which might reveal your identity, such as "*We have previously shown that….*[ref]" or "*Following on from our earlier studies….*[ref]". Alternative phrasing could be "*It has been shown previously that….*[ref]" or "*Earlier studies found that….*[ref]".
• Please avoid citing any of your own work in the reference list which has yet to be accepted for publication. If you believe such work would be useful or relevant to your submission, please provide this to the editor separately.
• For review-type articles, please include your author biographies in a separate file, not in the body of the main article.
• You should avoid signing responses to reviewers' comments and appeal rebuttals with author names.
• For revised manuscripts and resubmissions, please provide us with a 'double-anonymised title page' in .doc(x) form using the template provided. We require this to data capture the author names, affiliations, author contributions and acknowledgements for our production system; it will not be sent to reviewers.
• Please note that, for submissions which contain data in repositories, such as CIF files deposited with the CCDC, it is not possible to fully comply with double-anonymised peer review.

This checklist was updated on 25 May 2021.
